# Novel *TNC-PDGFD* fusion in fibrosarcomatous dermatofibrosarcoma protuberans: a case report

**DOI:** 10.1186/s13000-021-01123-1

**Published:** 2021-07-13

**Authors:** Yuan Chen, Ying-zhou Shi, Xiao-he Feng, Xiao-tong Wang, Xiang-lei He, Ming Zhao

**Affiliations:** 1 Department of Pathology, Laboratory Medicine Center, Zhejiang Provincial People’s Hospital, People’s Hospital of Hangzhou Medical College, 158 Shangtang Road, Gongshu District, Hangzhou, 310014 Zhejiang China; 2 Department of Pathology, Haining Central Hospital, Jiaxing, Zhejiang China; 3 Department of Pathology, Nanjing Jinling Hospital, Nanjing University School of Medicine, Nanjing, Jiangsu China

**Keywords:** Dermatofibrosarcoma protuberans, Fibrosarcomatous transformation, *TNC*, *PDGFD*, Fusion gene

## Abstract

**Background:**

Dermatofibrosarcoma protuberans (DFSP) is a superficial fibroblastic tumor characterized by high rate of local recurrence and low metastatic potential. Fibrosarcomatous transformation can rarely arise in DFSP either de novo or as recurrent, which represents a form of tumor progression and carries an increased risk of metastasis over classic DFSP. Cytogenetically, DFSP is characterized by a recurrent unbalanced chromosome translocation t (17;22)(q22;q13), leading to the formation of *COL1A1-PDGFB* fusion transcript that is present in more than 90% of cases. Alternative fusions involving the *PDGFD* with partners of *COL6A3* or *EMILIN2* have recently been documented in less than 2% of cases. Herein, we report a DFSP with fibrosarcomtous morphology harboring a novel *TNC-PDGFD* fusion.

**Case presentation:**

A 54-year-old female presented with a slowly growing mass in the right thigh. Excision demonstrated a 2-cm ovoid, well-circumscribed, gray-white, mass. Microscopic examination revealed a partially encapsulated subcutaneous nodule without dermal connection. The neoplasm was composed of cellular and fairly uniform spindle cells with brisk mitoses, arranged in elongated fascicles and herringbone patterns, with focal collagenized stroma. The neoplastic cells were positive for CD34 and smooth muscle actin. Fluorescence in-situ hybridization analyses showed negative for *COL1A1-PDGFB* fusion as well as *NTRK1/2/3* rearrangements. A subsequent RNA sequencing detected an in-frame fusion between exon 15 of *TNC* and exon 6 of *PDGFD*. This fusion was further confirmed by nested reverse transcription polymerase chain reaction amplification followed by Sanger sequencing. A diagnosis of fibrosarcomatous DFSP was rendered and the patient was in good status at a follow-up of 12 months after the operation.

**Conclusions:**

We report a fibrosarcomatous DFSP with novel *TNC-PDGFD* fusion, which adds to the pathologic and genetic spectrum of *PDGFD-*rearranged DFSP.

## Background

Dermatofibrosarcoma protuberans (DFSP) is a locally aggressive but rarely metastasizing, fibroblastic neoplasm that typically presents as a nodular and multinodular cutaneous mass on the trunk and proximal extremities of young to middle-aged adults [1, 2]. Classically, DFSP is composed of fairly uniform, mildly atypical spindle cells, often arranged in tight storiform, whorled, or cartwheel patterns. It usually originates in the dermal with subsequent infiltration the subcutaneous fat with a characteristic honeycomb appearance. By immunohistochemistry (IHC), the neoplastic cells usually express CD34 with focal expression of smooth muscle actin (SMA) sometimes observed [1, 2]. Fibrosarcomatous transformation can rarely arise in DFSP either de novo or as recurrent, which represents a form of tumor progression and carries an increased risk of metastasis over classic DFSP [3, 4]. The fibrosarcomatous component varies in proportion from less than 5% to more than 95%, and often arises in the subcutis with a nodular, rather well-demarcated growth pattern [3, 4]. The tumor cells in fibrosarcomatous areas often arrange in fascicular and herringbone-like and frequently have greater cytologic atypia, increased cellularity and mitotic activity than those in the ordinary DFSP. By IHC, fibrosarcomatous DFSP commonly exhibits diminished, even loss of CD34 expression and increased P53 expression [3, 4]. Cytogenetically, the vast majority of DFSPs (including those with fibrosarcomatous change) harbor the recurrent unbalanced chromosome translocation t (17;22)(q21;q13), commonly in the form of supernumerary ring chromosomes, resulting in the fusion of genes *COL1A1* on chromosome 17q21.3 and *PDGFB* on 22q13 [5, 6]. It has been proposed that constitutive expression of PDGFB is the fundamental mechanism of tumorigenesis in DFSP [5, 6]. However, rare variant fusion such as *COLIA2-PDGFB* fusion [7] and alternative rearrangements involving the related *PDGFD* gene with partners of either *COL6A3* or *EMILIN2* have also been documented recently [8, 9].

Herein, we report a fibrosarcomatous DFSP in which a novel fusion between *TNC* and *PDGFD* genes was detected by RNA sequencing and further confirmed by nested reverse transcription polymerase chain reaction (RT-PCR) amplification followed by Sanger sequencing .

## Case presentation

A previously healthy 54-year-old female presented with a slowly growing mass in the right thigh for 1 year. Enhanced computed tomography scan demonstrated a 2.2 cm, well-defined, subcutaneous nodule in the right thigh with moderately heterogeneous enhancement. The tumor was surgically removed with narrow margins. No evidence of tumor recurrence or metastasis was noted at a follow-up of 12 months after the operation.

**Fig. 1:**
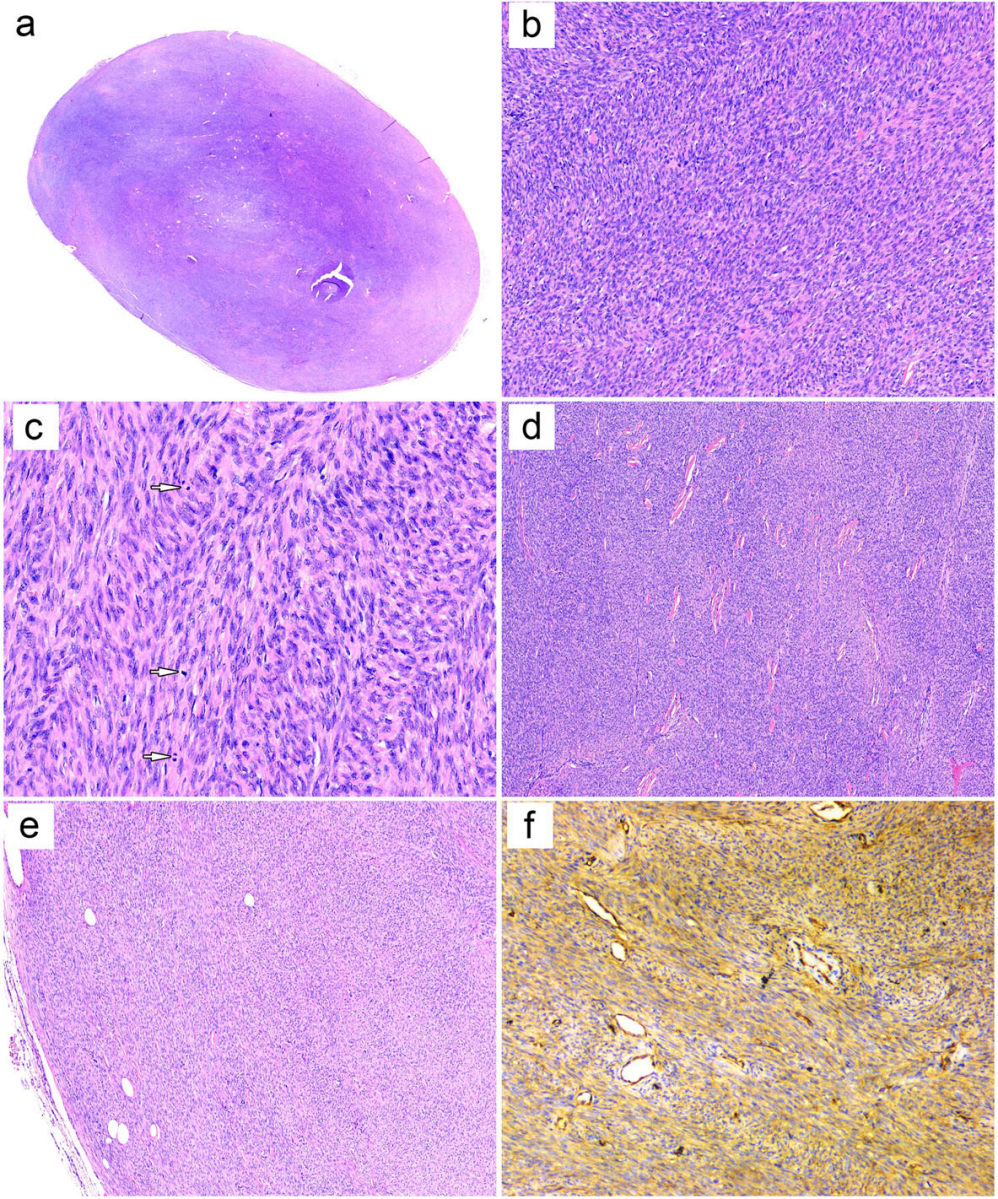
**a** Low-power view demonstrating a partially encapsulated nodular mass without dermal connection. **b**, **c** Cellular and mildly atypical spindle cells with brisk mitoses (*arrows*), arranging in elongated fascicles and herringbone patterns. **d** Focal collagenized stroma. **e** Minor areas displaying classic dermatofibrosarcoma protuberans with storiform growth and entrapment of adipocytes. **f** Immunoreactivity for CD34

Gross examination revealed a 2 cm, well-circumscribed nodular mass with a firm, gray-white cut surface. Low power magnification showed a well-defined, partially encapsulated subcutaneous tumor without dermal connection (Fig. 1a). The tumor was composed of cellular, fairly uniform, slightly rounded spindle cells containing scant cytoplasm and ovoid, mildly atypical nuclei with brisk mitoses (up to 10 mitotic figures per 10 high power fields). The tumor cells were arranged predominantly in elongated fascicles and herringbone architectures (Fig. 1b, c). The stroma was typical minimal with scattered round small vessels and focal depositions of thick and band-like collagen bundles (Fig. 1d). No tumor necrosis was noted. At the periphery of the mass, minor areas showing vague storiform growth of less cellular and more bland-appearing tumor cells setting in a more collagenized stroma, with occasionally entrapped mature adipose tissues, were observed (Fig. 1e).

**Fig. 2:**
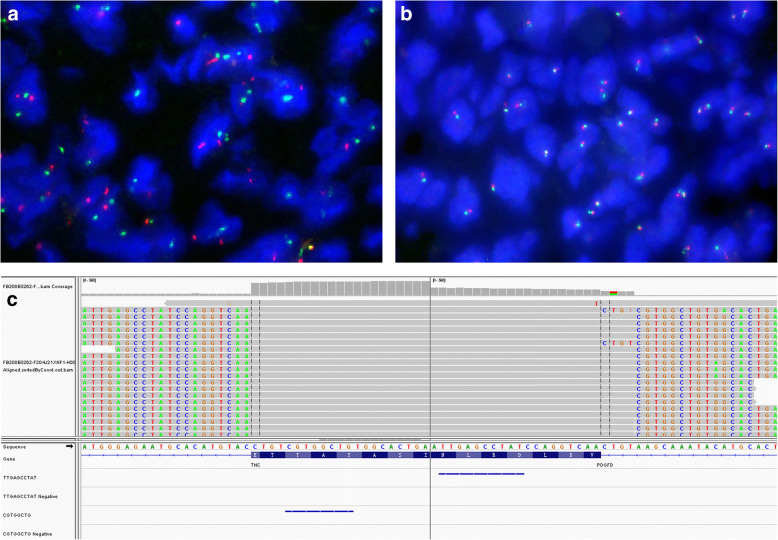
Fluorescence in-situ hybridization analyses revealing negative for (**a**) *COL1A1-PDGFB* fusion using the dual spanning probe set and (**b**) *NTRK1* rearrangement with the break-apart probe set*.* (**c**) RNA sequencing suggesting a chromosomal translocation of *TNC* gene exon 15 on chromosome 9 with *PDGFD* gene exon 6 on chromosome 11

Immunohistochemical staining revealed the tumor cells to be positive moderately and diffusely for CD34 (Fig. 1f) and focally for SMA and negative for Cam5.2, epithelial membrane antigen (EMA), desmin, calponin, TLE-1, CD99, Stat6, anaplastic lymphoma kinase (ALK, 1A4), SOX10, and S100 protein. The Ki67 proliferation index was approximately 20%. Fluorescence in-situ hybridization (FISH) analysis was negative for fusion of the *COL1A1* and *PDGFB* using the dual spanning probe set (Fig. 2a). Assessment for rearrangements of the *NTRK1/2/3* locus using the corresponding break-apart probe sets were all negative (Fig. 2b).

**Fig. 3:**
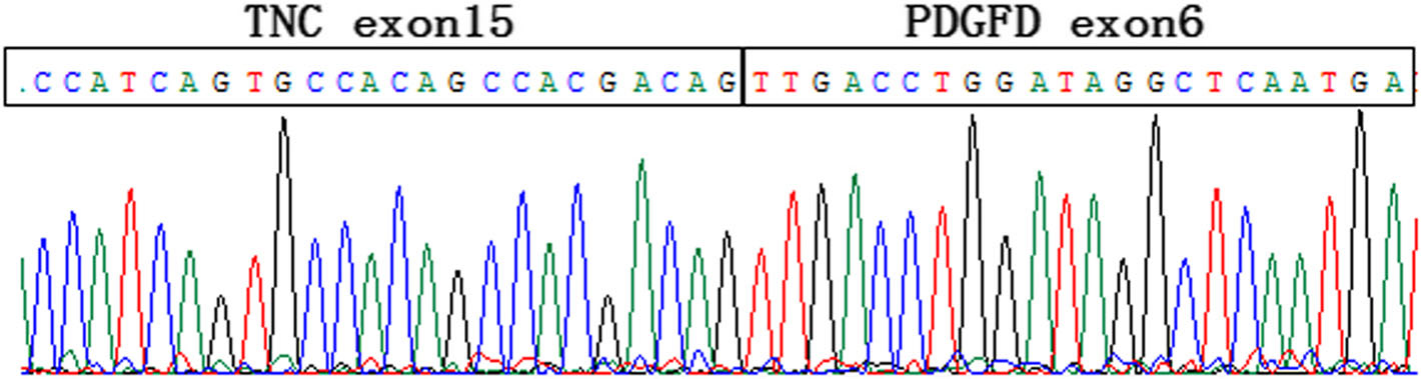
Sanger sequencing of the nested RT-PCR amplification product further confirmed the *TNC-PDGFD* fusion transcript

RNA sequencing was performed on formalin-fixed paraffin-embedded (FFPE) tissue as described previously [10]. Specifically, total RNA was extracted from FFPE samples with a RNeasy FFPE kit (QIAGEN). The quantity and quality of total RNA were assessed with the KAPA Library Quantification Kit (KAPA Biosystems), and the Agilent High Sensitivity DNA Kit and Bioanalyzer 2100 (Agilent Technologies), respectively, Sequencing was performed on the Illumina HiSeq next-generation sequencing platform (Illumina). The results were then analyzed using the BLAT aligner, Factera and Socrates, respectively, as previously described [10]. A fusion product between *TNC* exon 15 and *PDGFD* exon 6 with the variant allele frequency (VAF) of 85.37% was identified. The fusion result was confirmed through manually reviewing on the Integrative Genomics Viewer (Fig. 2c). This fusion was further confirmed by nested reverse transcription polymerase chain reaction (RT-PCR) amplification (*TNC* forward primer 5′-TGGCTACCGATGGGATCTTC − 3′ and *PDGFD* reverse primer 5′-CCGAGTAATTCCTGGGAGTGC-3′) followed by Sanger sequencing (Fig. 3).

## Discussion

Historically DFSP has genetically been featured by t (17;22)(q22;q13) translocation, leading to the fusion of the *COL1A1* gene with the *PDGFB* gene [5, 6]. The *COLIA1-PDGFB* fusion results in the constitutive up-regulation of *PDGFB* expression, leading to autocrine activation of PDGF receptor β (PDGFR-β) receptor tyrosine kinase signaling and consequently drives the tumorigenesis [2, 11]. These provide a rationale for targeted therapy with tyrosine kinase inhibitors (TKIs) for unresectable or metastatic DFSPs [12]. The *COL1A1-PDGFB* fusion has been detected in up to 96% of DFSPs and represents a quite useful tool for the differential diagnosis of DFSP with its mimickers [1, 2, 6, 7, 13]. With the application of more sensitive detection assays, the fusion incidence appears to increase and rare cryptic fusions and alternative rearrangements involving the related *PDGFD* gene have also been documented [7–9]. In the few cases of DFSP that were negative for *COL1A1-PDGFB* fusion through routine FISH analysis, two recent studies using Next generation sequencing found that 40% of cases indeed had the classical *COL1A1-PDGFB* fusion, while more than half of cases harbored a fusion between *PDGFD* and either *COL6A3* or *EMILIN2,* with the *COL6A3-PDGFD* fusion much more frequently encountered than the *EMILIN2-PDGFD* fusion [8, 9]. In this report, we describe a novel *TNC-PDGFD* gene fusion in a DFSP with fibrosarcomatous morphology, enhancing the genetic spectrum of DFSP.

*PDGFD*, located at 11q22.3, encodes a protein belonging to the same family of platelet-derived growth factor as PDGFB [14]. It has been proposed that PDGFD displays an oncogenic activity specifically through binding to and activating its cognate receptor PDGFR-β, and plays an important role in regulating tumor cell growth, migration, invasion, angiogenesis and metastasis by cross-talk with many signaling pathways in a wide array of malignancies [14, 15]. In *PDGFD*-rearranged DFSP, the reported genomic breakpoint was constantly located in exon 6, which retained the PDGF domain in a manner similar to rearrangements involving *PDGFB* [5, 8, 9]. *TNC*, also known as *Tenascin-C,* located at 9q33.1, is a member of tenascin gene family and encodes an extracellular matrix glycoprotein TNC with a spatially and temporally restricted tissue distribution [16, 17]. TNC is homohexameric with disulfide-linked subunits, and contains multiple EGF-like and fibronectin type-III domains [16, 17]. TNC has oncogenic properties through promotion of cell proliferation, migration and angiogenesis and its over-expression has been linked to a variety of malignancies [18]. Recently, fusions involving *TNC* have rarely been documented to occur in other neoplasms, including *TNC-NRG1* fusion in a non-small cell lung carcinoma [19] and in a papillary renal cell carcinoma [20], and *TNC-USP6* fusion in a primary aneurysmal bone cyst [21]. In these scenarios, *TNC* is functioned as a strong promoter, leading to activation of oncogenes *NRG1* and *USP6*, and subsequently induction of tumor formation.

According to the limited cases published to date, the *PDGFD-*rearranged DFSPs are commonly centered in subcutaneous fat without dermal involvement [8, 9], and the *COL6A3-PDGFD* fusion variant shows an apparent proclivity for the breast or chest wall locations in female patients and present with classic histology and immunophenotype [8], while both the two reported cases of DFSP harboring *PDFGD-EMILINE2* fusion arise in the leg and demonstrate a fibrosarcomatous morphology [9]. Studies from 2 groups by Dickson et al. [8] and Dadone-Montaudié et al. [9] have showed that *PDGFD*-rearranged DFSP clustered together with the group of DFSP with the classic *COL1A1-PDGFB* fusion upon unsupervised hierarchical clustering analysis, and demonstrated increased expression of *PDGFRB* mRNA by RNA sequencing. These evidence suggested that *PDGFD* rearrangements may function in a similar pattern of autocrine activation via PDGFR-β receptor tyrosine kinase signaling as *COL1A1-PDGFB* fusions, and rearrangements of *PDGFD* might therefore be targeted by TKIs as classical DFSP [8, 9].

Fibrosarcomatous DFSP represents morphological progression to a usually fascicular and herringbone pattern with increased risk of recurrence and metastatic potential [1–4]. The underlying oncogenic mechanism of fibrosarcomatous transformation in conventional DFSP is largely undetermined. Several different molecular genetic alterations have been proposed to account for this transformation, including genomic gains of *COL1A1-PDGFB*, losses of genomic material from 22q, mutations of *TP53,* activation of the PDGFR-β/Akt/mTOR pathway signaling, and microsatellite instability [22]. Most recently, single reports have suggested that over-expression of programmed cell death 1 ligand (PD-L1) [23] and fusion of *MAP 3K7CL-ERG* [24] may be implicated in the transformation of conventional DFSP to fibrosarcomatous DFSP. It’s worth noting that both the previously reported two cases of *EMILIN2-PDGFD* fusion DFSP exhibited a fibrosarcomatous histology and showed homozygous deletion of *CDKN2A* [9], which had also been identified in *PGDFB*-rearranged DFSPs and often observed in cases showing hypercellularity and fibrosarcomatous transformation morphology [25]. These suggest that, despite limited experiences, disruptions of the CDKN2A/CDK4/RB1 pathway may also represent an oncogenic mechanism in the clonal evolution of a subset of *PDGFD*-rearranged DFSP with fibrosarcomatous transformation.

The case we described here harbors a novel *TNC-PDGFD* gene fusion, as detected by RNA sequencing and nested RT-PCR, and shares some features with *EMILIN2-PDGFD* fusion DFSP, including deep-seated without dermal connection, fibrsarcomatous transformation morphology with focally abundant collagenous stroma. Similar to other reported *PFGFD* fusions, the PDGF receptors DNA binding domain of *PDGFD* is preserved in the currently documented *TNC-PDGFD* fusion [8, 9]. As with the *EMILIN2-PDGFD* fusion, it is difficult to determine with certainty if *TNC-PDGFD* plays a driving role in the transformation of this tumor from DFSP to fibrosarcomatous DFSP, but it is a possible candidate. Larger cohorts with functional studies will be needed to further assess the role of *TNC-PDGFD* and the behavior of DFSP containing this fusion. With regard to the differential diagnosis of this tumor, *NTRK*-rearranged tumors may be a consideration given the monomorphic spindle cells, CD34 expression, and focally sclerotic background. However, *NTRK*-rearranged tumors typically co-express S100 protein, often harbor *NTRK1* rearrangement although rare rearrangements involving *NTRK2*, *NTRK3*, *RAF1*, and *BRAF* have been documented [1] .

In summary, we report a fibrosarcomatous DFSP with novel *TNC-PDGFD* fusion, which adds to the pathologic and genetic spectrum of *PDGFD-*rearranged DFSP. The expanded molecular spectrum provides a novel insight into DFSP oncogenesis and carries important implications for molecular diagnostics as well as potential tailored therapies.

## Data Availability

Records and data pertaining to both the cases are in the patient’s secure medical records in Zhejiang Provincial People’s Hospital, People’s Hospital of Hangzhou Medical College. All searched data by literature review are included in this paper.

## Abbreviations

ALK: anaplastic lymphoma kinase
DFSP: dermatofibrosarcoma protuberans
EMA: epithelial membrane antigen
FISH: fluorescence in-situ hybridization
IHC: immunohistochemistry
PDGFR-β: PDGF receptor β
PD-L1: programmed cell death 1 ligand
RT-PCR: reverse transcription polymerase chain reaction
SMA: smooth muscle actin
TKIs: tyrosine kinase inhibitors
VAF: variant allele frequency
